# Exceptionally long‐lasting response to dabrafenib plus trametinib treatment in a patient with lung adenocarcinoma harboring the *BRAF* V600E mutation with high expression of PD‐L1: A case report

**DOI:** 10.1111/1759-7714.15254

**Published:** 2024-03-01

**Authors:** Takako Inoue, Kei Kunimasa, Motohiro Tamiya, Takahisa Kawamura, Toshiyuki Minami, Kazumi Nishino

**Affiliations:** ^1^ Department of Thoracic Oncology Osaka International Cancer Institute Osaka Japan; ^2^ Department of Respiratory Medicine and Hematology Hyogo Medical University Nishinomiya Japan; ^3^ Department of Thoracic Oncology Hyogo Medical University Kobe Japan

**Keywords:** *BRAF* V600E, case report, dabrafenib plus trametinib, immunotherapy, non‐small cell lung cancer

## Abstract

We present a patient with lung adenocarcinoma showing high PD‐L1 expression and *BRAF* V600E mutation, who achieved a remarkable long‐term response to the combination therapy of dabrafenib and trametinib (DT treatment) after disease progression on immunotherapy. This case may provide an opportunity for clinicians to consider the order of administration of immunotherapy and molecular targeted therapy for *BRAF* V600E‐positive lung cancer.

## Graphical abstract

The clinical course of DT treatment after immunotherapy.

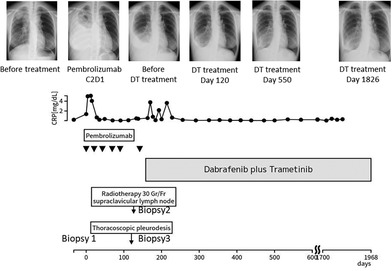

## INTRODUCTION

The *BRAF* V600E mutation, occurring in 1%–2% of lung adenocarcinomas, acts as an oncogenic driver. *BRAF* mutations cause downstream activation of the MAPK signaling pathway.^1^ First‐line treatment with the combination of BRAF/MEK inhibitors offers clinical benefits and is approved by the US Food and Drug Administration (FDA) and European Medical Agency.^2^

BRAF‐mutated non‐small cell lung cancers (NSCLCs) exhibit responsiveness to anti‐programmed cell death‐ligand (PD‐L) 1/PD‐L1 antibodies. In cases of BRAF V600E mutation,^1^ the efficacy of second‐line immunotherapy may surpass that observed in the overall population,^2^ with an objective response rate ranging from 20% to 30%. Guidelines advocate prioritizing molecularly targeted therapies for BRAF in the first‐line setting, irrespective of PD‐L1 expression status. Combinations of targeted therapy and immunotherapy have demonstrated clinically relevant activities. Nevertheless, limited data are available to guide the optimal timing and sequence of administering these treatments in patients with NSCLCs harboring the BRAF V600E mutation. Here, we present a patient with lung adenocarcinoma who showed high PD‐L1 expression and *BRAF* V600E mutation. The patient presented with disease progression at 6 months after anti‐PD‐1 antibody therapy but long‐lasting response (>5 years) on dabrafenib‐trametinib (DT) therapy.

## CASE REPORT

**FIGURE 1:**
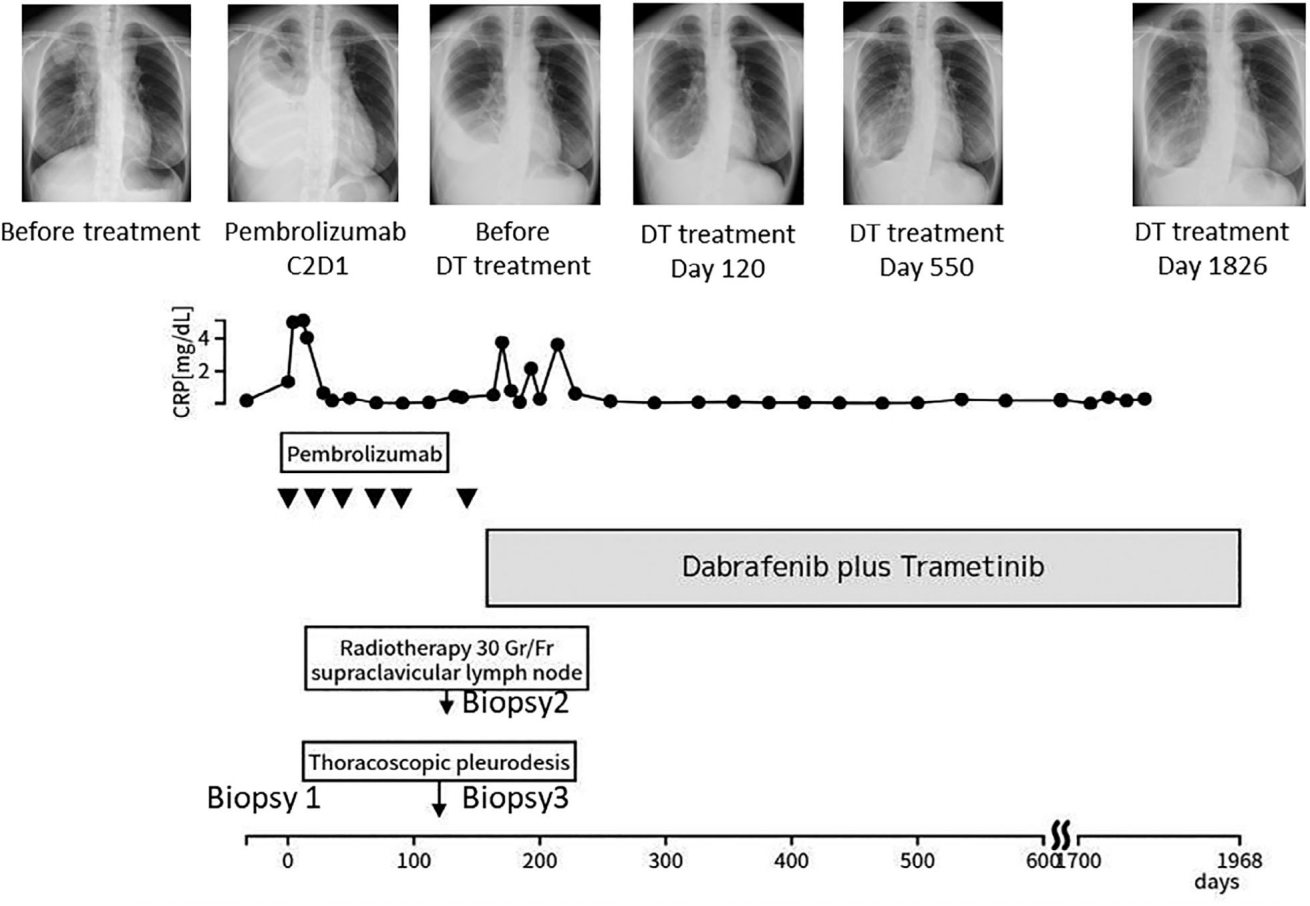
Clinical course. After initiating pembrolizumab therapy, the enlargement of lymph nodes from the neck to mediastinum and the right pleural effusion worsened rapidly. However, after 3 weeks, there was a trend of gradual improvement and the treatment was continued as the findings were assessed as pseudo‐progression. After 3 months, the right pleural effusion exacerbated and the subclavian and axillary lymph nodes re‐enlarged. Dabrafenib and trametinib (DT treatment) was administered as a second‐line therapy. One week after the initiation of dabrafenib‐trametinib (DT) therapy, axillary and subclavian lymphadenopathy alleviated. After 3 weeks, the increase in pleural effusion stopped.

A 30‐year‐old female nonsmoker presented with an abnormal chest shadow in the right upper lobe. There was no medical, family, and psychosocial history. Computed tomography (CT) revealed a nodule of diameter 20 mm in the right S1 and extensive swelling of the lymph nodes, which extended to a contralateral mediastinal lymph node and bilateral clavicle‐cervical lymph node. Histological examination of a sample collected via endobronchial ultrasound‐guided transbronchial needle aspiration was performed. The patient was diagnosed with T1cN3M0 stage IIIb lung adenocarcinoma with a programmed death ligand‐1 (PD‐L1) tumor proportion score (TPS) of 100%. A single polymerase chain reaction test was performed for *EGFR* and *ROS1*, and an immunohistochemical analysis was performed for *EML4‐ALK*, which tested negative. Chemoradiotherapy was not indicated due to the large extent of lymph node involvement in the bilateral supraclavicular fossa and the mediastinum. The patient was started on pembrolizumab 200 mg for 3 weeks as the first‐line treatment for an incurable stage IIIb NSCLC. The patient showed temporary enlargement of lymph nodes from the neck to the mediastinum and increased pleural effusion, but the treatment was continued because the tumor began to shrink after 3 weeks (pseudo‐progression) (Figure 1).

**FIGURE 2:**
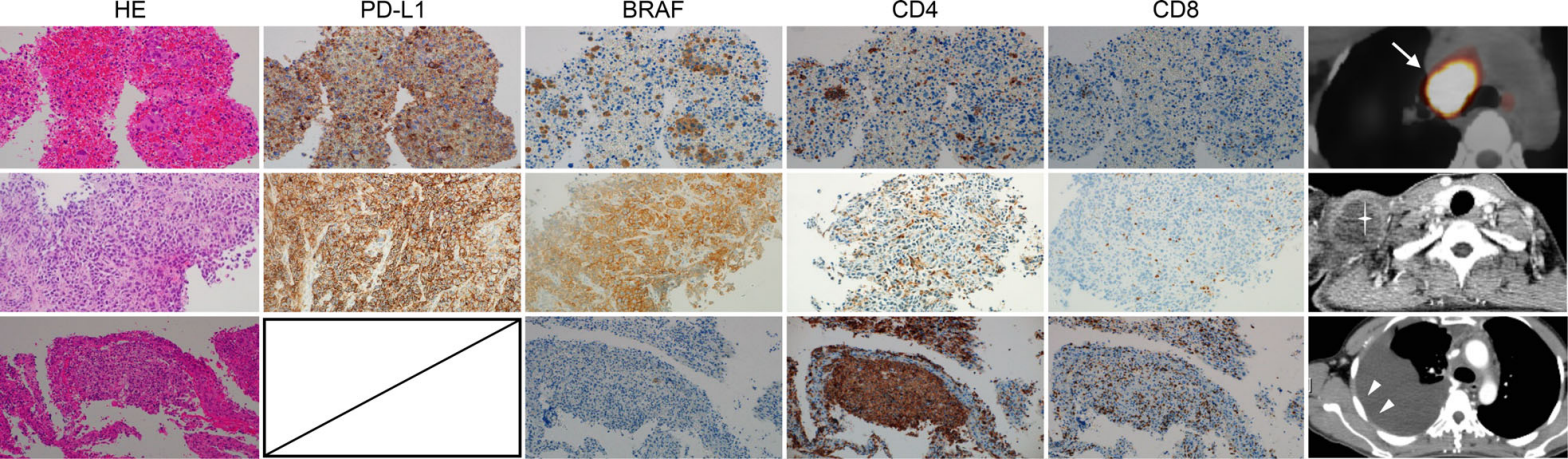
(a) Lymph nodes at the time of lung cancer diagnosis. In peripheral blood, tumor cells with irregularly sized nuclei and acidophilic cytoplasm were scattered in small clusters or as individual cells. The membranes and some cytoplasm of the tumor cells were highly positive for programmed death‐ligand 1 (PD‐L1) (clone 22C3; Dako) (TPS ≥50%). The cytoplasm of the tumor cells was diffusely positive for BRAF (clone V600E; Roche). The surrounding inflammatory cells were diffusely positive for PD‐L1. CD8‐positive lymphocytes were observed bordering the tumor cells, although they were scarce. (b) Pleural lesions after immunotherapy. The pleura showed reactive hyperplasia, was coated with stratified mesothelial cells, and had marked inflammatory cell infiltration consisting mainly of numerous foamy macrophages and lymphocytes. The ratio of CD4‐positive to CD8‐positive lymphocytes was 10:7, representing a relative increase from that before treatment. Histomorphologically, residual tumor cells were difficult to identify, with only 3–4 cells weakly positive for *BRAF* and some degenerating tumor cells present in solitary sections. PD‐L1 (22C3) could not be determined because of the paucity of the tumor cells. (c) Cervical lymph nodes enlarged after immunotherapy. Tumor cells that were polymorphous with hyperchromicities, ubiquitous nuclei, and weakly acidophilic cytoplasm were found to have infiltrated in an irregular focal, tissue‐destructive manner, with fibrous growth patterns. The cytoplasm of the tumor cells was BRAF‐positive. The membranes and some tumor cell cytoplasm were also highly positive for PD‐L1 (clone 22C3; Dako) (TPS ≥ 95%). There was a marked increase in the number of CD4‐positive lymphocytes compared to the lymph node involvement at the time of diagnosis; however, the increase in CD8‐positive lymphocytes was limited compared to what was observed in the pleural lesions.

At 3 months after starting pembrolizumab, the patient had enlarged lymph nodes and showed markedly increased pleural effusion again. Because the pretreatment pleural effusion was cytologically negative, a pleural biopsy by thoracoscopy under local anesthesia was performed. Histological examination of the pleura revealed a small number of tumor cells with marked tumor‐infiltrating lymphocytes. Histological examination of the subclavian lymph nodes showed tumor cell proliferation with mild lymphocytic infiltration. (Figure 2). The patient developed neck pain and facial swelling, and pembrolizumab treatment was assessed to be ineffective. To reduce cervical lymph node pain and facial edema, radiotherapy at 30 Gr/Fr was administered to the supraclavicular lymph nodes.

Analysis using the Oncomine Comprehensive Assay version 3 (Thermo Fisher Scientific Inc.) revealed the presence of the *BRAF* V600E mutation. Next‐generation sequencing (NGS) liquid biopsies; Guardant360 CDx (NGS LBx) identified coexpression of *BRAF*/TP53 mutational status. Thereafter, the patient was administered dabrafenib 150 mg twice per day plus oral trametinib 2 mg once per day as a second‐line therapy. After DT initiation, pleural effusion stopped, and a marked reduction was noted in lymph node involvement. Fever (grade 2), elevation in aspartate aminotransferase/alanine aminotransferase (grade 2) ratio and γ‐glutamyl trans peptidase level (grade 3), and erythema nodosum of the extremities (grade 2) were observed as adverse events of DT treatment; however, these improved with temporary steroid use.

**FIGURE 3:**
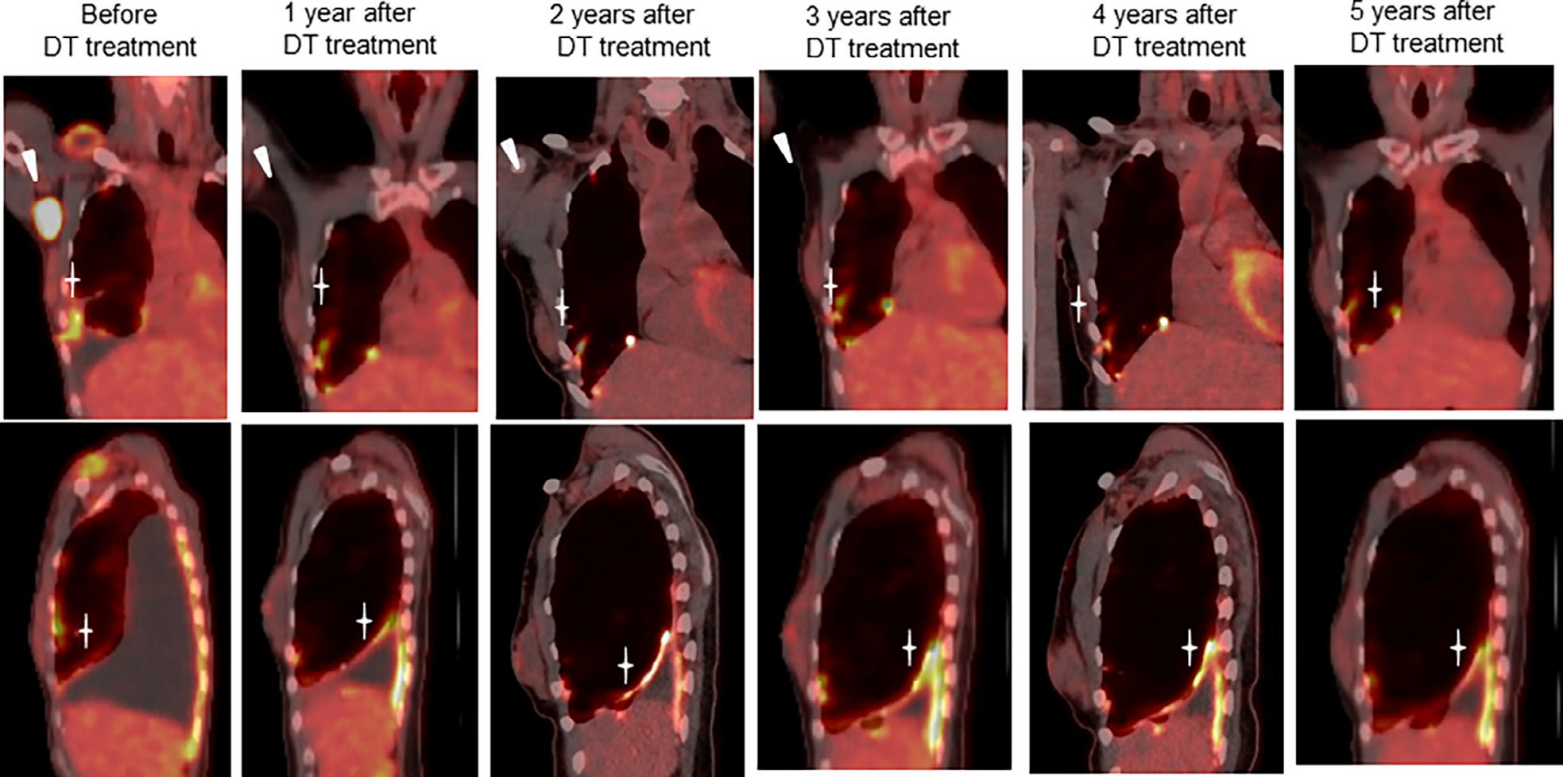
18F‐fluorodeoxyglucose positron emission tomography (18F‐FDG‐PET) images before the start of dabrafenib‐trametinib (DT) treatment showed strong accumulation of FDG in the axillary lymph nodes and diffuse accumulation of FDG on the pleura. After 1 year of treatment, the abnormal accumulation in the axillary lymph nodes disappeared, but the diffuse pleural accumulation repeatedly worsened and decreased.

At the time of writing this report, that is 5 years since the patient's initial visit, DT treatment was successful, and annual 18F‐fluorodeoxyglucose positron emission tomography (18F‐FDG‐PET) scans revealed pleural inflammation. There have been no trends indicating tumor progression (Figure 3).

## DISCUSSION

In our patient, pembrolizumab treatment was ineffective, with biopsies of each lesion showing viable tumor cells and clinical symptoms indicative of disease progression. As the first course of the therapy, we could not choose chemoradiotherapy, because the contralateral mediastinal metastases and bilateral clavicle‐cervical metastases were extensive and not within the range of radical radiation therapy. In addition, combined immunotherapy and chemotherapy was not an option because it had not yet received approval in Japan.

In this case, the patient received pembrolizumab monotherapy, but we chose chemoimmunotherapy because of the high response rate, which could have prevented early progression of disease with immunotherapy.

On subsequent DT treatment, the patient showed an exceptionally long‐term response (>5 years) compared with that previously reported for similar cases. This could be partially attributed to the activation of antitumor immunity by pembrolizumab. For patients with lung adenocarcinoma harboring the *BRAF* V600E mutation, there is no clear evidence for the choice of treatment (i.e., DT treatment or standard first‐line treatment for patients without targetable driver alterations).

Five‐year updates from a phase II clinical trial showed a median progression‐free survival (PFS) with DT treatment of 10.2 months (95% confidence interval [CI]: 6.9–16.7) in 57 pretreated patients with *BRAF* V600E‐mutated NSCLC and 10.8 months (7.0–14.5 months) in 36 treatment‐naïve patients.^3^ This result suggests the efficacy of DT, regardless of the treatment line. Although *BRAF* V600E‐harboring lung adenocarcinoma tends to show higher PD‐L1 expression, there are no comprehensive reports on the efficacy of first‐line immunotherapy for lung cancers harboring the *BRAF* V600E mutation. In our case, the tumor exhibited primary resistance to first‐line pembrolizumab. Subsequent DT treatment was effective for a long period. Annual 18F‐FDG‐PET scans showed continued inflammation of the pleura, and a pleural biopsy taken at the time of pembrolizumab failure showed strong lymphocyte infiltration—suggesting that the activation of tumor immunity by pembrolizumab may have contributed to the long‐term response to DT. The combination of atezolizumab with vemurafenib and cobimetinib has been reported to substantially improve PFS compared to vemurafenib and cobimetinib alone in cases of advanced malignant melanoma. Therefore, the possible use of DT and immunotherapy combination for lung cancers harboring the *BRAF* V600E mutation merits further investigation.

Recently, a phase III trial of immunotherapy versus DT for advanced malignant melanoma harboring *BRAF* V600E reported considerable improvement in the 2‐year survival rate when immunotherapy was administered as the first‐line therapy. Intriguingly, prior immunotherapy did not seem to influence efficacy of DT (in lung cancer supported by the Planchard et al. study with similar ORR first‐/further line) while resistance to DT appeared to modulate response to immunotherapy.^4^ The remarkable efficacy of immunotherapy in NSCLC characterized by *KRAS*/TP53 mutations and PD‐L1 high expression (>50%) has been documented.^5^ In this case, coexpression of *BRAF*/TP53 mutational status and PD‐L1 high expression may be linked to prolonged immunotherapeutic efficacy.

It may even be preferred over molecularly targeted agents as the first‐line therapy for lung cancers with high PD‐L1 expression harboring targetable driver mutations of components of the MAPK pathway, including *KRAS* and *BRAF*.^6^

The coadministration of immunotherapy and molecular‐targeted drugs, or the prompt initiation of molecular‐targeted drugs shortly after concluding immunotherapy, is recognized for its potential to elevate complications.^7^, ^8^, ^9^ Sotorasib, when combined with pembrolizumab or atezolizumab for advanced KRAS p.G12C NSCLC, was associated with a heightened incidence of grade 3–4 treatment‐related adverse events in comparison to immunotherapy alone, notably liver enzyme elevations.^8^, ^9^ In this instance, the occurrence of grade 3 liver injury was also attributed to molecular targeted therapy following immunotherapy, underscoring the importance of vigilant side‐effect management when employing combination therapy.

In conclusion, patients with lung cancer harboring the *BRAF* V600E mutation and showing high PD‐L1 expression, immunotherapy might represent the first‐line treatment of choice, considering the tumor volume and performance status. This case suggests that first‐line immunotherapy may contribute to the long‐term efficacy of subsequent DT therapy. Thus, when managing NSCLC cases harboring mutations in genes encoding components of the MAPK signaling pathway, clinicians should consider the treatment sequence.

## AUTHOR CONTRIBUTIONS

**Takako Inoue:** Conceptualization, Data curation, Investigation, Writing‐original draft, Writing‐review and editing. **Kei Kunimasa:** Conceptualization, Investigation, Writing‐review and editing. **Motohiro Tamiya:** Investigation, Writing‐review and editing. **Takahisa Kawamura:** Investigation, Writing‐review and editing. **Toshiyuki Minami:** Investigation, Writing‐review and editing. **Kazumi Nishino:** Conceptualization, Investigation, Writing‐review and editing.

## CONFLICT OF INTEREST STATEMENT

The authors declare the following financial interests/personal relationships that may be considered as potential competing interests. Dr Inoue reports honoraria for lecture fees from AstraZeneca, Ono, MSD, and Chugai. Dr Kunimasa reports honoraria for lecture fees from AstraZeneca, Chugai Pharma, and Novartis. Dr Tamiya reports receiving grants from Boehringer Ingelheim, Ono, MSD, Eisai, Daiichi Sankyo, Chugai, and Janssen; and personal fees from Boehringer Ingelheim, Ono, MSD, Chugai, AstraZeneca, Taiho, Eli Lilly, Novartis, Asahi Kasei, Bristol‐Myers Squibb, Bayer, Amgen, Kyowa‐Kirin, and Nippon Kayaku. Dr Nishino reports receiving grants from Ono, TAIHO, MSD, AbbVie, DAIICHI SANKYO, Amgen, Eisai, Sanofi, Janssen, Novartis, Pfizer, Eli Lilly, Merck, Takeda, Chugai, and Merus; and personal fees from AstraZeneca, Chugai, Nippon Boehringer Ingerheim, Eli Lilly, Roche, Novartis, Pfizer, Merck, Janssen, Bristol Myers Squibb, and Nippon Kayaku. The remaining authors declare no conflict of interest.
